# Humic Substances as a Versatile Intermediary

**DOI:** 10.3390/life13040858

**Published:** 2023-03-23

**Authors:** Simona Hriciková, Ivona Kožárová, Nikola Hudáková, Anna Reitznerová, Jozef Nagy, Slavomír Marcinčák

**Affiliations:** 1 Department of Food Hygiene, Technology and Safety, University of Veterinary Medicine and Pharmacy, 04181 Košice, Slovakia; 2 Centre for Experimental and Clinical Regenerative Medicine, University of Veterinary Medicine and Pharmacy, 04181 Košice, Slovakia

**Keywords:** humic substances, humic acids, fulvic acids, human health, animal health, environment, One Health

## Abstract

Humic substances are organic ubiquitous components arising in the process of chemical and microbiological oxidation, generally called humification, the second largest process of the carbon cycle. The beneficial properties of these various substances can be observed in many fields of life and health, whether it is the impact on the human organism, as prophylactic as well as the therapeutic effects; animal physiology and welfare, which is widely used in livestock farming; or the impact of humic substances on the environment and ecosystem in the context of renewal, fertilization and detoxification. Since animal health, human health and environmental health are interconnected and mutually influencing, this work brings insight into the excellence of the use of humic substances as a versatile mediator contributing to the promotion of One Health.

## 1. Introduction

Humic Substances (HSs) are well-known, high-molecular-weight, heterogenous organic compounds. They are present in a wide range of environments. HSs are refractory compounds that arise by the action of microbial metabolism as the end products of organic decomposition in marine, freshwater and soil environments [1,2,3]. In further detail, HSs are products of profound chemical and microbiological oxidation of precursors such as lignin, tannins, peptides, cellulose, lipids and terpenoids from plant and animal remnants. Additionally, in the carbon cycle, humification is regarded as the second-largest process after photosynthesis [1,4].

The International Humic Substances Society (IHSS), which was established in the United States (Denver, Colorado) in 1981, serves as the connecting force for HSs. IHSS was created to bring together scientists with interests in coal, soil and water. It has close to 900 scientists as members. The Society is regarded as a global leader in advancing academic inquiry and research as well as raising public awareness of humic compounds. The motto of the IHSS is: “To Advance the Knowledge and Research of Natural Organic Matter in Soil and Water” [5]. One of the goals of the Society is to build and maintain a collection of standard samples of humic acids (HA) and fulvic acids (FA) from lignite, fresh water, mineral soil and organic soil, as well as to collect characterization data. The Society has also included reference samples that serve as a source of HSs for study. International conferences organized by the IHSS every two years bring together experts in the fields of soil, coal, freshwater and marine sciences [5].

Several authors reviewed the effectiveness of the use of HS in many areas in recent years. HSs, as representative of natural organic matter and the most common organic compounds existing in the environment, has been applied to the treatment and remediation of environmental pollution [1]. HA are promising green materials for water and wastewater treatment. They show a strong ability to sorb cationic and hydrophobic organic pollutants. Chianese et al. (2020) reviewed and discussed the interaction between organic pollutants and HA dissolved in solution, in the solid state and also adsorbed onto solid particles, like aluminosilicates and magnetic materials. They additionally included a short discussion of the thermodynamics and kinetics of the sorption process of HA [6]. HSs presence alters biological transformations of organic pollutants in both water and soil. HSs including Hu have a great potential for application in aerobic and anaerobic wastewater treatment as well as in bioremediation. The results of many original and review papers presented and discussed in this article show enormous potential for interesting, multidisciplinary research as well as for the development of new, green technologies for biological wastewater treatment and bioremediation [7]. Another study compared existing techniques for removing HA from wastewater, as well as their limitations. Physicochemical treatments including filtration and oxidation are basic and key approaches to removing HA. Biological treatments including enzyme and fungi-mediated HA degradation are economically feasible but require some scalability. The integrated treatment processes are more significant options for the effective removal of HA from wastewater. In addition, HA have rich utilization values. It can improve the soil, increase crop yields and promote the removal of pollutants [8]. The paper of Kudryasheva and Tarasova (2015) considered mechanisms of detoxification of pollutant solutions by water-soluble HSs, natural detoxifying agents. The detoxifying effects of HSs were shown to be complex and regarded as ‘external’ (binding and redox processes in solutions outside the organisms) and/or ‘internal’ organismal processes. They demonstrated that the HSs can stimulate a protective response of bacterial cells as a result of: 1. changes in rates of biochemical reactions and, 2. stabilization of mucous layers outside the cell walls [9].

The biomass was directly used extensively in agriculture due to its low cost and convenience. Increasingly serious soil pollution of heavy metals may pose a threat and risk to human health. Direct addition of biomass to soil may affect the bioavailability and content of heavy metals [10]. An article by Yang et al. (2021) reviewed the regulation mechanisms of biochar and HSs on phosphorus availability and circulation. This included improving soil physicochemical characteristics, regulating microbial community structure and directly interacting with phosphorus to affect the fate of phosphorus in soil [11]. The applications and working principles of such HSs in agriculture and environmental ecology, mainly to elaborate the multiple roles of this functional polymer along with physical–chemical quantification were collated and summarized by Yang et al. (2021) [1].

Pham et al. (2021) summarized all the available information on the properties of Hu as an extracellular electron mediator function, its applications, possible redox-active structures and the interaction between Hu and microbial cells [12]. The review of Peng et al. (2022) systematically introduces and summarizes the redox activity of HSs for the remediation of environmental pollutants. For inorganic pollutants (such as silver, chromium, mercury and arsenic), the redox reaction of HSs can reduce their toxicity and mobilization, thereby reducing the harm of these pollutants to the environment. With regards to organic pollutants, HSs have photocatalytic activity and produce a large number of reactive oxygen species under the light which reacts with organic pollutants to accelerate the degradation of organic pollutants. Finally, the research direction of HSs redox remediation of environmental pollution is prospected [13].

HSs are dominant components of soil organic matter and are recognized as natural, effective growth promoters to be used in sustainable agriculture. In recent years, many efforts have been made to gain insight into the relationship between HSs chemical structure as well as their biological activity in plants using combinatory approaches. Relevant results highlight the existence of key functional groups in HSs that might trigger positive local and systemic physiological responses via a complex network of hormone-like signalling pathways [14]. The review written by Tiwari et al. (2022) offers insight into the various structural and functional aspects of HSs, particularly the HA. The dynamic and interactive nature of HSs creates the framework of sustainable agriculture [15]. As declared in a review by Ma et al. (2022), current knowledge revealed that HSs have great potential to elicit plant tolerance to various abiotic stresses, thus enhancing plant growth and performance-related parameters (such as root growth/diameter, flowering, nutrient use efficiency/translocation, soil water holding capacity and microbial activity) [16]. Another paper labelled HSs as biostimulants for terrestrial photosynthetic organisms. Their effects on plants are related to specific HSs features such as: pH and redox buffering activities, (pseudo)emulsifying and surfactant characteristics, capacity to bind metallic ions and to encapsulate labile hydrophobic molecules, ability to adsorb to the wall structures of cells [17]. Da Silva et al. (2021) state that HSs are promising options for reducing the use of pesticides and mineral fertilizers [18]. The review of Nardi et al. (2021) gave a detailed survey of the mechanisms associated with the growth regulatory functions of HSs in view of their use in sustainable technologies [14]. A review by Cristofano et al. (2021) presented a comprehensive look at the scientific literature regarding the widely used and EU-sanctioned biostimulant substances including HSs. Starting from their origin, the modulation of plants’ hormonal networks, physiology, stress defence systems and their in vivo effects are all discussed with regard to some of the most prominent vegetable species of the popular plant groupings of cucurbits, leafy greens and nightshades. The review concluded by identifying several research areas relevant to biostimulant substances to exploit and enhance the biostimulant action of these substances and signalling molecules in horticulture [19]. Baltazar et al. (2021) declared that HSs have a proven potential in improving plant growth, increasing crop production and quality, as well as ameliorating stress effects [20]. In relation to HSs effects on soil fertility, Garcia et al. (2019) discussed the relationships between two main signalling pathway families that are affected by HSs within the plant: one directly related to hormonal action and the other related to reactive oxygen species [21]. Shah et al. (2018) discussed various regulatory activities and distribution strategies of HSs on soil fertility and crop productivity owing to their unique physiochemical and biochemical properties, as well as the establishment of biotic and abiotic interactions within the plant rhizosphere [22].

The use of HSs in medicine has been included in the following studies: Yilmaz and Dizman (2023) reviewed the causes of *Varroa destructor* infestations, the applied control methods and the applicability of HA as an antiparasitic agent [23]. Traditional medicine and modern research claim FA can modulate the immune system, influence the oxidative state of cells and improve gastrointestinal function; all of which are hallmarks of diabetes. Examples of successful development of humic-based sorbents for fluoroquinolone and tetracycline removal from environmental water systems or polluted wastewater were given in another review by Kulikova et al. (2022). Data on the various effects of HSs on the dissemination of antibiotic-resistance genes (ARGs) were summarized. The detailed characterization of HSs properties as a key point of assessing the environmental consequences of the formation of antibiotic-HS complexes, such as the dissemination of antibiotic resistance, was proposed [24]. Due to the very widespread and important topic of ARGs, the review described the main mechanisms by which HSs affect the ARGs evolution includes electrostatic interactions, hydrophobic interactions, hydrogen bonding, non-specific van der Waals interactions and microbial community alterations. Finally, future research directions are proposed to help refine the theoretical basis for the interaction between HSs and ARGs. These can contribute to the prevention and control of antibiotic pollution in practical applications [25]. The minireview of Winkler and Ghosh (2018) outlines the available peer-reviewed research on FA and examines its anecdotal health claims. They showed that, although available research has been minimal, there is substantial evidence to pursue FA research in preventing chronic inflammatory diseases, including diabetes [26]. Another clinical review presented what is known about the antiviral features of HSs to the benefit of the clinical healthcare provider using available data in humeomics, the study of the soil humeome. It provides the reader with a working framework of historical studies and includes clinically relevant data with the goal of providing a broad appreciation of the antiviral potential of humic substances while also preparing for a translational leap into the clinical application of HA [27]. Arif et al. (2019) described the useful applications and recent facets of HA including its modes of action and various valuable uses in improving the production and health safeguarding of livestock and poultry [28].

Many industries, including agriculture, ecological restoration, environmental protection, animal husbandry and aquaculture, veterinary medicine and human medicine, have discovered crucial uses for HSs as fertilizer enhancers, bonding of metals, agricultural bio stimulants and antifungal activity [29,30,31,32,33,34,35]. When using them, we can also argue with the idea of One Health, because the potential of HSs can be used in many different contexts, as in 2021 One Health was defined by the One Health High-Level Expert Panel (OHHLEP) as “an integrated, unifying approach that aims to sustainably balance and optimize the health of people, animals and ecosystems. It recognizes that the health of humans, domestic and wild animals, plants and the wider environment (including ecosystems) are closely linked and interdependent” [36]. When the health of one is at risk, the health of all may be at risk.

Given the excellence of HSs in supporting the health and well-being of humans, animals and the environment, the aim of this manuscript is to provide a reader with the current knowledge about: (a) the HSs from a chemical perspective, including their structure, extraction and the relationship between their physical, chemical and biological properties and uses; (b) the impact of HSs on the human health, animal physiology and the environment; (c) selected supplements and preparations of HA and FA currently commercially available on the market.

In this review, we do not focus solely on one effect of HSs, but we have understood their properties from a broad perspective based on the One Health concept. In spite of the fact that is important to conduct research focused on individual areas of action of HSs, our article combines all aspects of the use of HSs in the complex of life as such. As we focus on the broad-spectrum effects of HSs, throughout this study we describe the effects of HSs on human, animal and environmental health, which together form a single whole. In addition to summarizing the great properties of HSs for individual subjects, we also provide lists of commercially available products containing HA and FA that can be used to improve human, animal and environmental health. The benefit of our article lies in the comprehensiveness of the information focused on all spheres of action of HSs and also the available preparations applicable to it.

## 2. Material and Methods (Data Search)

In this study, we performed a narrative review searching original published papers on humic substances, including the data filter on humic substances, extraction of humic substances, effect on humans, effect on animals, effect on the environment and both medical and agriculture use. Criteria included a published scholarly peer-reviewed journal from the last two decades, without a language or country restriction. The review protocol included a Google and a Google Scholar search along with four databases, PubMed^®^, Web of Science^®^, Scopus^®^ and ScienceDirect^®^.

General information search: For obtaining general information about humic substances, we conducted a search using Google Scholar, PubMed^®^ and Web of Science^®^. Keywords used: “humic substances” OR “humic acids” OR “fulvic acids” AND “source” OR “extraction” OR “characteristics” and related searches. There were no restrictions for language or country; however, we restricted searching for articles written in the last five years.

Study search: For obtaining information and results from in vitro, in vivo or clinical trials, we carried out our search using PubMed^®^, Web of Science^®^, Scopus^®^ and ScienceDirect^®^. We searched for studies written in the last 20 years with the following keywords: “humic substances” OR “humic acids” OR “fulvic acids” AND “therapy” OR “health” OR “anti-inflammatory” OR “absorbent” OR “environment” and searches related to those published in the last twenty years.

Search for commercially available products containing humic substances: Humic substances based products currently available on the market intended for humans, animals and agriculture were searched for using Google search. The research was performed without any language or country restrictions including the criteria: humic substances, humic/fulvic acids, humic/fulvic capsule/powder, humic/fulvic humans humic/fulvic animals, humic/fulvic agriculture/soil/water/plants and related searches. From all the products found, those containing HA or FA as main ingredients were selected. From the results found, a sample of products from different manufacturers, different countries of origin, composition and purpose of use were selected.

Data extraction: The following information was chosen: author(s), publication year, nation, study goal, study design and key findings. The data was extracted by three separate researchers, who also chose a sample of papers that qualified, with a high agreement. The articles were arranged first by title and summary, then by full text. Articles that were duplicates or did not fit the search parameters were removed.

More than 5000 articles were initially found, out of which only 75 were selected for drawing information. The selected articles were separated based on the criteria mentioned above to ensure they were suitable for the content of our review. Data collection was completed in January 2023.

## 3. Composition, Extraction and Dimension of Humic Substances

The specific composition of HSs may differ due to their origin and region of occurrence [37]. However, the commonly recognized fundamental components of HSs include; humins (Hu), HA and FA [37,38,39]. Hu, HA and FA occur naturally in the form of amorphous substances ([3]. Hu complexes are categorized as macro organic compounds due to their large molecular weight. Hu are black in colour. They are insoluble at all pH values because they are tightly linked to inorganic soil colloids. The Hu percentage boosts the soil’s ability to hold onto water. The molecular size of HA ranges from 10,000 to 1,000,000. Colour of HA varies from dark brown to grey and black [3,37,38,39]. Although humic acid is not directly toxic, it may produce undesirable colour, taste and odour in drinking water [40]. HA are either odourless or having a slight petroleum-like smell [41], as another study on the pungent odour of extracted HA reported [42]. As they are composed of a combination of carbon rings and chains, HA are insoluble in water in an acidic environment. However, they are soluble in alkaline conditions and precipitable at pH < 2. In comparison to HA, FA have a significantly smaller molecular size which ranges from 1000 to 10,000. The colour of FA varies from light yellow to yellow-brown and they are odourless. FA are mostly composed of carbon, hydrogen, oxygen and nitrogen. They are soluble in water at all pH levels. Proton breakdown in FA results in a gradual array of negative charges as pH increases. It is now well documented that carboxylic type groups are mostly to blame for this behaviour across the lower pH range, such as those below pH 7. The primary sources of FA are polysaccharides and low molecular weight fatty acids [3,37,38,39].

Because of HSs’ complexity, irregularity and formation pathways, they should not be regarded as rigidly defined molecules, but rather as comprising a range of characteristics. The two most significant of these characteristics are: (1) the presence of ionic structures, such as carboxylic and phenolic groups, which affect humic matter solubility and lead to complex formation of metals and other toxins; and (2) the incidence of aromatic structures, which absorb light, trigger a variety of photochemical processes and are involved in sorption process and aggregation [43].

To identify the individual structural units of HSs, the research on HSs composition has far been carried out, using strong oxidants (alkaline solution) [14,44,45], first used in 1786 [46]. The use of an alkaline solution, sodium hydroxide (NaOH) and/or potassium hydroxide (KOH) for the extraction of humus allows the dissociation of interactions (hydrogen bonds, metal bridges-bonds) between organic matter and different soil components [21] and is common in most methods still in use [47,48,49].

**Figure 1:**
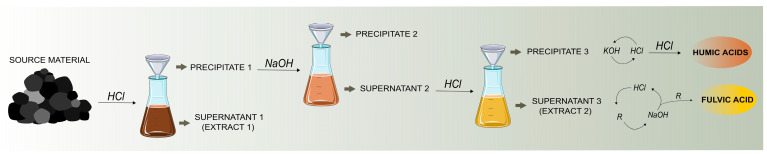
Graphical representation of the extraction of Humic and Fulvic acids from solid-phase source materials of Humic substances. HCl—hydrochloric acid; NaOH—sodium hydroxide; KOH—potassium hydroxide; R—resin treatment. The Figure was created using Inkscape software and Smart.servier database.

IHSS describes a generally successful standardized procedure of extractional isolation of HSs (HA and FA) from solid-phase source materials (Figure 1), taken from Swift 1996. Pre-treatment of the sample consists of acidification using hydrochloric acid (HCl). This step is helpful to remove part of carbonate. The suspension is then separated into the supernatant 1 (supernatant 1, extract 1) and the precipitate (precipitate 1) by decantation after allowing the solution to settle or by low-speed centrifugation and filtration. Extract 1 (containing FA) is saved for later. The precipitate (precipitate 1) is treated with an alkaline solution (NaOH) which leads to the formation of the precipitate (precipitate 2; an insoluble fraction, contains Hu) and the supernatant (supernatant 2), which are separated by centrifugation and filtration. Supernatant 2 is acidified with HCl and separated by centrifugation into precipitate (precipitate 3, containing HA) and supernatant (supernatant 3, extract 2, containing FA). The HA fraction (precipitate 3) is then purified in a cycle of dissolution (KOH) and precipitation (HCl). To get FA, extract 1 and extract 2 are mixed and passed through a column (resin treatment—R), followed by elution (NaOH) and acidification (HCl) [50].

Numerous studies and credible documentation have been carried out on the HSs elemental composition. In summary, the IHSS standard and reference FA and HA included the following amounts of different components; organic carbon (C; 50–60%), hydrogen (H; 3.5–4.8%), nitrogen (N; 0.7–5.1%) and oxygen (O; 31.6–45.5%) [50,51,52]. The pedo-climatic conditions and anthropogenic activities have a significant impact on the elemental composition and, as a result, the functional groups [52]. The bioactivity of HSs structures associated with their complexity has both been extensively discussed [4,14,17,20,53,54,55]. The theories of HSs molecular and supramolecular structures have been established in recent investigations. The revelation lies in the fact that humic compounds have substantially lower molecular masses than previously thought [56,57].

## 4. The Influence of Humic Substances on the Human Health

HSs are effective as dietary supplements, as they appear to have only a few side effects (and even those only in highly limited dietary conditions) [58]. HSs designated as the biologically active additives used in medical and veterinary medicine practice are positioned as enterosorbents, immunomodulators [59,60,61] and bioregulators of a new generation [62,63]. The immunomodulatory effect of HA was demonstrated by the course administration of HA isolated by the sodium pyrophosphate method from pine-sphagnum-cotton sedge peat reduced the general anaphylaxis reaction in mice and guinea pigs immunized with ovalbumin and decreased serum content of IgG1 and IgE in mice. The serum from mice treated with HA sensitized with ovalbumin did not increase the rate of degranulation of mast cells isolated from intact Wistar rats in the presence of ovalbumin in comparison with the serum of control animals [60].

HSs have a variety of physiological impacts on organisms, including modulating stress responses [64,65,66,67], having hormone-like effects [68,69], regulating genes, activating various signal transduction pathways through interactions with membranes and regulating ion exchange [37,70].

In both people and animals, HSs have been shown to have anti-inflammatory [71], antibacterial, antiviral [72] and anticarcinogenic effects [73]. The ability to activate the metabolisms of water, protein and fat as well as their bactericidal effect make the low molecular-weight fractions of HA useful for treating gastrointestinal, skin and other illnesses [62]. Several studies have been conducted on the antiviral activity of HSs, severally anti-human immunodeficiency virus type 1 [4,74,75], anti-COVID-19 [37,76] and anti-herpes simplex virus type 1 [77]. The antiviral activity of HSs against COVID-19 disease was studied by Hajdrik et al. (2022). HSs containing ascorbic acid, Se^−^ and Zn^2+^ ions intended as a nutritional supplement material were investigated against SARS-CoV-2 virus B1.1.7 Variant of Concern (“Alpha Variant”) in VeroE6 cell line. Results showed that this combination has a significant in vitro antiviral effect at a very low concentration range of its intended active ingredients. Even picomolar concentration ranges of HSs, vitamin C and Zn^2+^/Se^−^ ions, in the given composition were enough to achieve 50% inhibition of a viral replication in the applied SARS-CoV-2 virus inhibition test. This antiviral effect can be caused by the components’ additive effects on the cell-virus system (e.g., antioxidant effect, reduction in viral replication, enhancement of the resistance of cell membrane, reduction in inflammatory mediator cytokine secretion, etc.) in a synergistic manner [37].

By observing the formation of micronuclei (MN), leonardite humic acids (LHA), soil humic acids (SHA), fungicidal activity of HA isolated from an empty fruit bunch of oil palm compost (EFB-HA) and commercial grade humic acids (Co-HA) were evaluated on the conidial germination and mycelial growth of Choanephora cucurbitarum. EFB-HA and Co-HA significantly (*p* ≤ 0.05) inhibited the mycelial growth and conidial germination of C. cucurbitarum compared to the control. The inhibition in mycelial growth increased with an increase in HA concentrations tested, suggesting the presence of fungicidal activity. The maximum inhibition of conidial germination (71.96% after 24 h) was exerted by EFB-HA at the highest concentration (1000 mg·L^−1^). It was concluded that the efficiency of HA studied in controlling fungal growth was apparently related not only to their concentration and the pathogen examined, but also to HA origin and nature and, in particular, HA structural and functional properties, especially COOH group content [78].

In animals, HSs show antimutagenic activity, immunomodulating and antioxidant effects. The antimutagenic effect of processed HA was studied using the yeast strain *Saccharomyces cerevisiae* D7 test. Results showed that the antimutagenic effect of HA was strongly dependent on their preparation procedure. The highest antimutagenic activity was observed when using simple sodium and potassium humates [79]. Antimutagenic/desmutagenic activity of a leonardite humic acid (LHA) and a soil humic acid (SHA) was investigated in the cultured human lymphoblastoid cell line TK6 treated with mitomycin C (MMC) as a reference mutagen by evaluating the induction of micronuclei (MN). Both HA dramatically decreased the frequencies of MN generated by MMC. The two HA also showed a modest cell-protective effect in the desmutagenic test against the cytotoxicity of MMC. The larger carboxylic group content and lower phenolic group concentration of LHA may be the reason for its stronger antimutagenic/desmutagenic action compared to SHA. These findings support the antigenotoxic activity of HAs in human cells, which is consistent with earlier findings in a diverse range of plant species [80].

Antioxidant activity of HSs has been shown in recent studies [62,81,82]. It was demonstrated by Khiľko et al. (2011) that the model reactions of started radical chain oxidation of hydrocarbons are successfully inhibited by the significant antioxidant capabilities of HA from brown coal (cumene and ethylbenzene) [62]. HA can be suggested for use as potential naturally occurring antioxidants that are physiologically active for the creation of new classes of medications for use in medicine. Antioxidant and anti-apoptotic effects of HA compared with the histopathological and neurological outcomes for hypoxic-ischemic brain injury were investigated. Results showed that HA reduces apoptosis and neuronal injury in the cerebral tissue of rats. These findings suggest that HA may be an active protective agent against hypoxic-ischemic encephalopathy [81].

Since the potential of HSs to act preventively and curatively against many diseases has been used we can find many dietary supplements suitable for humans (Table 1). The most common form of supplements are capsules, but also suspensions, which have higher bioavailability and provide more convenient administration.

## 5. The Effect of Humic Substances on the Physiology of Animals

According to scientific research, bacterial resistance is encouraged by the use of antibiotics at concentrations below therapeutic levels in animal production. Antibiotic-resistant bacteria have been found in greater quantities in ecosystems, slaughterhouses and faecal samples. Antibiotic-resistant microorganisms may negatively impact human health [83]. As a result, in 2006, the European Union totally outlawed the use of growth-promoting antibiotics in animals [84,85]. Similarly, the World Health Organization’s suggestions on the use of medically necessary antimicrobials in animal production for human consumption advise against using any antibiotics at all to promote growth in food animals for human consumption [83,86]. It is important to consider other approaches in order to decrease the need for medications to maintain and safeguard animal health. As has been seen thus far, HSs can provide answers in many areas of health, not just for animals.

The unique properties of HSs allow them to be a useful and multifaceted ingredient in animal feeds. Farmers and researchers have been feeding and testing HSs since the early 1930′s [87]. Humic matter is being used in a variety of feed additives to help animals develop and thrive, sometimes even taking the place of antibiotic performance enhancers [37]. HSs can be used in a wide range of animal species as feed additives, with great benefit, especially in farm animals. For the list of feed supplements suitable for individual animal species see Table 2 below.

It has been demonstrated over the past 20 years that adding HA to poultry feed or water in a predetermined proportion may enhance growth performance and meat quality [88]. Numerous publications claimed that HA’s capacity to alter intestinal physiology is the basis for its positive impact on growth performance. Its inclusion in the poultry diet can stabilize the intestinal microbiota and hence improve digestibility and nutrient assimilation [89]. A recent study confirmed the improvement in growth performance shown in broiler chickens administered HA may be due to an increase in helpful lactic acid bacteria and a decrease in the proliferation of detrimental parasites in the chickens’ guts [89]. In addition to its beneficial impact on the digestive tract ecosystems, by enhancing haematological and biochemical parameters and fostering immunity, HA can help birds to grow [90,91]. Maguey-González et al. (2022) proved that HA can be used as a prebiotic in poultry as well [83].

The most recent experiment demonstrated the impact of HSs and humic-fatty substances (HFS) in a granular compound feed on the performance of fattening, the quality of meat and chosen biochemical measures in rabbit blood serum. The lipid and mineral parameters were favourably affected by HSs or HFS supplementation, which was shown by decreased total cholesterol and higher levels of calcium (Ca) and phosphorus (P) (*p* 0.05) in the blood serum of both supplemented groups. These findings allow us to predict that adding humic-fatty compounds to rabbit diets will enhance blood biochemical parameters and meat quality indicators [92]. The final meat quality of broiler chickens, which was favourably impacted by increased protein, decreased fat content and showed lower lipid oxidation was the major benefit of the broiler's diet supplemented with HSs [93,94]. HSs not only have a positive effect as a growth promoter, but can also reduce the mortality of farm animals [95].

A study of FA showed that the addition of 500 mg·kg^−1^ FA may boost production and egg quality and modulate the caecal microflora abundance and serum biochemical indices of laying hens [96].

Humates block or reduce the production of stress-causing hormones, improving animal behaviour. Humate-fed animals are less aggressive and least affected by outside disturbances like heavy crowds or traffic or during confinement in closed arenas [64,65,66]. The effect of HSs has also been demonstrated in calf breeding. HSs effectively improved the immune status, antioxidant capacity and intestinal beneficial bacteria, further improved the growth performance and reduced the diarrhoea incidence of the pre-weaned dairy calves [97].

## 6. The Impact of Humic Substances on the Environment as a Whole

Humic compounds are now seen as a potential development in green chemistry as a readily available and affordable source of raw materials for the production of commodities with significant chemical value [98]. Natural and modified humic preparations are promising as surfactants [99], polymers with adjustable redox properties (redox polymers) [13,100], and the base for the preparation of new drug preparations, chemical weed and pest killers, new sorbents [101] and preparations for the recultivation of territories contaminated by radionuclides, heavy metals, organic eco-toxicants, petroleum products, etc. [62].

In natural systems, HSs interact with microbial communities, dissolved natural organic matter, clays and minerals to inactivate and degrade human-made synthetic toxins (pesticides, polycyclic aromatic hydrocarbons, polychlorinated aliphatic hydrocarbons, petroleum) and deactivate toxic metals (arsenic, mercury, cadmium) [102]. FA can be used to bind heavy metal ions from soil or water because of their high affinity towards metal ions and unique chemical spatial structure [103,104,105,106]. The interactions of HSs with toxic pollutants mainly consist of absorption, binding, association, sequestration and entrapment of the toxic molecule by the massive humic molecules [102]. 

The majority of elements that compose soil organic matter are made up of HSs, which are also the most prevalent organic materials in the environment. These substances are complex combinations of organic waste that have undergone biological transformation [107]. Due to the role of HSs in many complex chemical and biochemical reactions in soils, HSs largely determine soil quality, and they are vital to maintaining soil fertility [50,108]. In Agriculture, the range of humic products includes dry granular products, liquid products and powders. Humic products are widely used as soil amendments and/or blended with fertilizers [109].

About 50% of the dissolved organic molecules in aquatic systems, such as rivers, are HSs, which have an impact on pH and alkalinity. The chemistry, cycle and bioavailability of chemical elements, as well as the transport and degradation of xenobiotic and naturally occurring organic compounds are all impacted by HSs in terrestrial and aquatic systems. Along with formation, they have an impact on the biological productivity of aquatic habitats [7,50]. HA, which are present in water, contribute to its pollution by changing the colour of the water and accelerating the creation of harmful disinfection by-products, such as toxic chemicals from chlorination, during the water treatment process. Although they exhibit poor polyelectrolyte behaviour, they may still react with substances while being resistant to degradation [110,111,112,113]. Dissolved HSs have a long-term impact on the physical and chemical characteristics of water and can serve as the most significant natural purifying agents. They have the ability to control the bio-concentration of toxins as well as serve as a source of organic nutrients in the short term [108].

It has been investigated that HA have a high affinity to polar, ionic pesticides of high water solubility, which are bound via specific interactions with HA functional groups. Studied biochar, due to its moderately hydrophobic character, preferentially attracts non-ionic pesticides of relatively high logP values and low water solubility. Hydrophobic bonding is postulated as a main mechanism of attraction. Besides sorbent structural properties, pH is the main factor governing sorption equilibria in the studied mixtures [114].

Due to all these beneficial properties of HSs, there is a huge number of products containing HA/FA on the market. These, with an emphasis on the ecosystem, can be employed as fertilizers, soil quality improvers, adsorbents, biostimulants, detoxifiers, litter additives, etc. The list of selected products can be found in the Table 3. 

## 7. Conclusions

If HSs have a positive impact on a wide range of issues related to human, animal and ecosystem, then we can talk about the idea of One Health. This is an idea that unites the interests of several areas and acts in the interest of good for all involved. The approach mobilizes multiple sectors, disciplines and communities at varying levels of society to work together to foster well-being and tackle threats to health and ecosystems, while addressing the collective need for clean water, energy and air, safe and nutritious food, taking action on climate changes and contributing to sustainable development. The versatile potential of HSs stems from the fact that they are substances of natural origin that also affect objects of natural origin and therefore organisms and components of the ecosystem. Nowadays, when there are countless pills and various preparations on the market and all around us, it is advantageous to discover an effective natural ingredient whose abilities can be applied to various purposes. In a broad sense, we can describe HSs as “an easily available solution of organic origin” for widespread application. Despite numerous studies to date, many of the effects of HSs have not yet been studied. We believe that future research will reveal other spheres of action of these all-rounders.

## Figures and Tables

**Table 1:** The list of selected dietary supplements containing humic substances intended for humans.

Product Name	Content (mg)			Application Form	Purpose (Based on Manufacturer Information)	Producer	Country of Origin
	HA	FA	Hu				
Detox Fulvic/Humic Foot Soak	800	50	-	powder	Helps absorb minerals; destroys toxins; supports the immune system; improves and softens the skin, good for blood circulation; for the care of tired muscles.	The Natural Edge	Australia/Canada
FULVIC	75	53	-	capsule	Rich source of natural minerals; traces elements, fulvic, humic and amino acids used for mineral supplementation that supports general health.	The Natural Edge	Australia/Canada
Fulvic Minerals	-	25	-	suspension	Supports gut health; strengthens immune function; boosts nutrient absorption; optimizes cellular function; improves energy levels; supports brain health; fights free radicals; supports detoxification.	Touchstone Essentials	USA
Fulvic/Humic Minerals, Inner Votality	2.2	8.3	-	suspension	Increases energy; improves vitality; mineral supplement.	Morning Star Minerals	USA
HUMAC^®^ Nativ	460	-	180	capsule	Absorption of toxins; anticarcinogenic effect; antioxidant activity; effect on iron deficiency, macro, micro and trace elements supplementation.	HUMAC^®^	Slovakia
Humic Acid Membrane Active	375	-	-	capsule	Immune system support; defends the body against toxins and foreign invaders and encourages a healthy immune response.	Allergy Research Group^®^	USA
HUMICIN Pure	50	75	-	capsule	Strengthens the immune system; reduces inflammation.	Humicin^®^	Hungary
HUMINIQUM	50	120	-	capsule	Antioxidant effects; significantly strengthens the immune system; has antiviral effects, binds toxins; helps with hair loss; helps with obesity; helps with fatigue, helps with skin problems; increases enzyme activity; increases permeability of cell walls; acts against anaemia, energizing effects; support regeneration after diseases; help with osteoporosis.	HYMATO PRODUCTS Kft.	Hungary
HUMINIQUM Sirup	20	48	-	syrup	Antioxidant effects; significantly strengthens the immune system; has antiviral effects, binds toxins; helps with hair loss; helps with obesity; helps with fatigue; helps with skin problems; increases enzyme activity; increases permeability of cell walls, acts against anaemia; energizing effects; support regeneration after diseases and helps with osteoporosis.	HYMATO PRODUCTS Kft.	Hungary
Jarrow Shilajit Fulvic acid complex	-	250	-	capsule	Works with Co-Q10 in the mitochondria to support energy production in brain and muscle tissues during exercise.	Jarrow Formulas	USA
Mineral Blend Fulvic-Humic	2.2	0.5	-	suspension	Supports a normal strong immune system; promotes digestive well-being; healthy cognitive function; restful sleep and assists the body’s normal ability to detoxify tissues.	Vital Earth Minerals	USA
Mumijo	60	250	-	capsule	Has a positive effect on the gastrointestinal tract; immune system; bones; fertility and brain.	Natu Gena	Germany
Refined Fulvic Acid	-	900	-	powder	Helps to absorb and use other nutrients such as microbiota/probiotics, phytonutrients, fatty acids and minerals; powerful natural free radical scavenger and antioxidant; neuroprotective; antimicrobial and anti-inflammatory properties.	Mark Nature	China
Trace Minerals Ionic Fulvic Acid	-	250	-	suspension	Helps support and maintain healthy functions in the body; including the following: digestion, pH balance, energy production, hydration, cellular integrity, enzyme activity, muscle endurance and stamina.	Trace Minerals	USA

HA = Humic acids; FA = Fulvic acids; Hu = Humins; Values of HA, FA, Hu are expressed in; mg·mL^−1^ (suspension and liquid form), mg·g^−1^ (powder form) and mg/capsule (capsule form).

**Table 2:** Humic substances based products available on the market intended for administration to animals.

Product Name	Content [%]			Form	Target Species	Purpose (Based on Manufacturer Information)	Producer	Country of Origin
	HA	FA	Hu					
HUMAC^®^ Natur AFM	65	5	-	powder	dog, cat	Supports the normal functioning of the immune system; promotes good health of the digestive system; supplements proper nutrition with minerals and trace elements; positively affects the condition, stress and appearance of pets; supports the normal functioning of the metabolism; supports the cleansing of the body from harmful substances.	HUMAC^®^	Slovakia
HUMAC^®^ Natur AFM Liquid	15	5	-	suspension	all animal species	Supplement in the treatment of various health problems, such as inflammation, digestive disorders, allergies, skin diseases, etc.; they improve the absorption of nutrients and minerals (a factor improving feed conversion); detoxification of the body; maintains various normal bodily functions such as immunity, digestive health, stable pH, normal stress levels, etc.	HUMAC^®^	Slovakia
HUMAC^®^ Natur—veterinary preparation	62	-	-	powder	all animal species	Detoxification of exogenous and endogenous poisons; stabilization of intestinal and rumen microflora; supports of the immune system; potentiation of the effects of probiotics, activation of metabolism; prevention of digestive disorders (diarrhoea).	HUMAC^®^	Slovakia
HUMAC^®^ Natur AFM for birds	65	5	-	powder	birds	Supports the normal functioning of the immune system; supports good digestive system health supplements the proper nutrition of birds with minerals and trace elements in chelate bonds has a positive effect on the condition, appearance/plumage/and stress of the birds; supports the normal functionality of the metabolism; supports the cleansing of the body from harmful substances.	HUMAC^®^	Slovakia
HUMAC^®^ Natur AFM Liquid for bees	15	5	-	suspension	bees	Supports the normal functioning of the metabolism of bees and protects them from metabolic disorders; binds toxins, other toxic or sprayed substances, PCB, dioxins and heavy metals, etc., which are subsequently eliminated by the digestive system; supports the normal functioning of the immune system and limits the occurrence of inflammation; supplements the proper nutrition of bees with minerals and trace elements; positively affects the condition and appearance of bees.	HUMAC^®^	Slovakia
HUMAC^®^ Natur AFM Liquid for horses	15	5	-	suspension	horses	To help in the treatment of various acute health disorders, such as food poisoning, weakened immune system, loss of appetite, etc. and used for prevention.	HUMAC^®^	Slovakia
HUMAC^®^ Natur—veterinary preparation for horses	62	-	-	powder	horses	Detoxification of exogenous and endogenous poisons, heavy metals and toxins in animal feed (microbial, fungal toxins, ammonia, PCBs, dioxins, bacterial toxins, viruses...); stabilization of intestinal microflora, prevention of digestive disorders, activation of metabolism; support of the immune system, affects the condition and appearance of the animals, health of the hooves, reduction of stress and general well-being of the animals; supplements the proper nutrition of horses with minerals and trace elements in chelate bonds.	HUMAC^®^	Slovakia
HUMAC^®^ Multi LISAL	6.5	-	-	salt lick	horses, donkeys, cattle, sheep, goats, ratic animal	Preventively protects animals from metabolic disorders (mainly diarrhoea), limits the occurrence of inflammation and supports the immune system; stabilizes the pH in the digestive tract, positively influences the activity and composition of the intestinal microflora, stimulates the production and activity of pancreatic enzymes; binds microbial toxins, fungal toxins and other toxic substances (ammonia, PCBs, dioxins, pesticides, heavy metals, etc.), which are then excreted in the faeces from the animal’s body.	HUMAC^®^	Slovakia
Moorkur liquid	20	-	21	powder	dogs, cats, birds, rodents, horses, small animals	Undeclared.	NatuVerde UG	Germany
HumicFed	60–70	10–20		powder/flakes	poultry, fish	Forms a somewhat protective film on the mucosa cells of the intestine; reduces the absorption of toxic substances; has the distinct property to absorb toxins from proteins, toxic residues and various heavy metals; stabilizes the intestinal flora, fixes micro-organisms, toxins and harmful substances in animal feed promote growth and stimulate the immune system.	KHUMIC	China
Sodium Humate	70	.	.	powder	poultry	Forms a kind of protecting film on the mucosa cells of the intestine; reduces the absorption of toxic substances; has the distinct property to absorb toxins from proteins, toxic residues and various heavy metals; stabilizes the intestinal flora; fixes micro-organisms, toxins and harmful substances in animal feed; promotes growth and stimulate the immune system.	Mark Nature	China
PrimeHumic	50	-	-	powder	farm animals	Promotes gut health; promotes the development of the intestinal structure; protects the intestine against harmful influences; promotes vitality; promotes the absorption of nutrients; improves overall health; helps to remove heavy metals and toxins.	BioAg Europe	Netherlands
PrimeHumic Liquid	52	12	-	liquid	farm animals	Promotes the immune system; nourishes and protects skin and fur; nourishes and protects skin and fur; promotes the absorption of nutrients; promotes intestinal health; improves feed conversion; less intestinal infection.	BioAg Europe	Netherlands
PrimeFulvic	-	6	-	liquid	farm animals	Promotes feed uptake; reduces stress level, enhances strength and energy; supports overall animal health.	BioAg Europe	Netherlands

HA = Humic acids, FA = Fulvic acids, Hu = Humins.

**Table 3:** Selected products containing humic substances for use in agriculture, aquaculture, as fertilizers, soil enhancers and adsorbents.

Product Name	Content [%]	Form		Purpose (Based on Manufacturer Information)	Producer	Country of Origin
	HA	FA				
Bio Fulvic Acid	-	95	powder	Enhances drought resistance of crops; increases activity of various enzymes and content of chlorophyll; regulates and stimulates crops to grow, increase their quality and yield 10–15%; foliar fertilizer; flush fertilizer; chemical pesticides additives, has obvious synergism.	Mark Nature	China
Boost Fulvic	-	3	liquid	Foliar spray; low pH liquid fertilizer; micronutrient enhancer.	Sustain Seed + Soil	USA
FULVAGRA^®^ Liquid 25	1	17	liquid	Increases the germination of seeds and supports root development; acts as natural complexing agent and increases fertilizer use efficiency in the soil and promotes nutrient uptake by plants; stimulates vigorous root development; increases the yield and improves plant quality; increases the cation exchange capacity in soil; stimulates enzymatic activity and increases the plant’s own defences against abiotic stress factors; promotes the collection and transport of micronutrients contained in leaf fertilizers; acts as an antioxidant against free radicals and thus prevents cellular damage.	Humin Tech	Germany
GHP DRY HP—POWDER	85	-	powder	Improves soil structure; improves the quality of soil; aids nutrient uptake; enhances the growth of soil organisms; improves nutrient uptake by plants.	Global Humic Products Ltd.	Canada
GHP LIQUID F—LIQUIFIED FULVIC	-	1.5	liquid	Improves nutrient uptake by leaves.	Global Humic Products Ltd.	Canada
HS^®^-300BIO BASIC	22	6	liquid	Improves fertilizer efficiency; promotes soil structure; reduces soil erosion; increases and secures yields and qualities; increases biomass production; binds CO_2_ in soil.	Humin Tech	Germany
HUMAC^®^ Agro	62	-	powder	Binds toxic metals, PCBs, dioxins, pesticides, herbicides and other toxic compounds in the soil; reduces the content of toxic substances in plant production products; supplies organic carbon to the soil; retains water in the soil (1 kg of preparation retains up to 740 mL of water); improves soil structure; adjusts soil pH; reduces the need for mineral fertilizers by up to 50%; improves the use of mineral substances from the soil by up to 70%; supplies the soil with all naturally occurring macro, micro nutrients and trace elements; supports the growth and reproduction of soil bacteria and useful soil microorganisms; significantly increases the growth of the root system of plants, thereby increasing the yield of cultivated plants and soil fertility.	Humac^®^	Slovakia
HUMAC^®^ Welfare	45	5	powder	For use in bedding and manure; binds emission gases (ammonia, methane, hydrogen sulphide, carbon dioxide), residues of toxic substances, microbial poisons, fungal toxins and other compounds poisonous to the organism (PCBs, dioxins, heavy metals, residues of pesticides, herbicides, etc.); protects housed animals from diseases, especially damage to the mucous membranes of the respiratory tract and conjunctivae; reduces hoof diseases; limits the hatching of fly larvae; significantly reduces the humidity of the litter; adjusts the pH of manure to acidic, or neutral (up to pH 7), when ammonia in manure occurs mainly in the form of a stable ammonium ion (NH4+), while in an alkaline environment (pH above 7) it quickly changes to volatile ammonia; significantly enriches manure, poultry droppings, etc. 0 nutrients contained in humic acids and nitrogen by absorption of ammonia; creates a high-quality, highly effective fertilizer with a natural stimulator of soil fertility, thereby improving the economic effect for the farmer in both animal and plant production; suitable for large farms: cattle, pigs, poultry, sheep, goats, rabbits, birds, small animals.	Humac^®^	Slovakia
Humasol Liquid Humic Acid Extract	6	-	liquid	For use in organic agriculture and safe for all crops including vegetables, herbs, nuts, fruits, berries, ornamentals, trees and shrubs.	Humasol	USA
Humic Fulvic Acid	60	55	powder	Soil conditioner; improves soil structure; increases ion exchange capacity of soil; fertilizer efficiency promoter; enhances nutrients uptake and increases the content of humus in soil; prevents soil from contamination of heavy metallic ions as well as other harmful matters; enhances solubility in hard water; plant growth stimulant; promote root development and stimulates seed germination; enhances nutrient uptake by combining nutrients and humic acids and keep well-balanced nutrition; enhances the resilience of crops (cold, drought, pest, disease and toppling resistance); increases yield and improves quality of plants; improves the effectiveness of pesticides.	Mark Nature	China
HumicFed	60–70	10–20	powder/flakes	Removes toxic metals and their ions from wastewaters; an adjunct in dissolved air flotation cells to assist in the removal of trace amounts of grease, oil, liquid organics and suspended matter; a coagulant aid to be used in conjunction with water-soluble polymeric flocculants for removal of soluble organics; fluid loss additives in certain types of organic liquids to prevent seepage from lagoons or pit containment areas.	KHumic	China
HumiFirst WG	53	12	powder	Powerful root stimulant and increases root length and biomass; increases the quantity of secondary (hair) roots that absorb nutrients and water; increases plant resistance to hydric and drought stress; improves the physical, chemical and biological characteristics of soil; increases aggregation of soil; increases recovery of nutrients, in particular phosphorous; increases soil wettability; increases root/shoot growth; increases microbial activity in soil; immediates efficacy and greater speed of action compared to other soil conditioners.	Tradecorp	Spain
Humistar	12	3	liquid	Plants: increases white roots improves nutrient uptake and plant vigour; improves flowering; increases resistance to disease; soil: better uptake of phosphorus and micro elements; increases the cation exchange capacity of soil, resulting in less waste of applied nutrients; improves soil structure which enables better aeration of soil and increases soil water holding capacity.	Tradecorp	Spain
KHUMIC-100	70	5–10	powder/flakes	Improves the structure of soil; increase the buffering power of soil; optimizes nutrients absorption by plants; neutralizes both acidic and alkaline soils, regulates the pH-value of soils, with the prominent effect in alkaline and acidic soil; reduces the toxins such as the ally aluminium and heavy metals; reduces high osmotic pressure within the root area; stimulates plant growth, increase the yield and quality; stimulates the membrane of seeds as well as the metabolic activities and thereby increase the germination rate; increases the capacity of root to take up nutrients; enhances cell assimilation; increases photosynthesis; reduces nitrate leaking into the groundwater and protect the underground water; increases the effectiveness of herbicide, pesticide and fungicide; immobilizes or reduce their harmful residues; stabilizes nitrogen and improve nitrogen efficiency (ideal as an additive with urea); complexes phosphate to reduce lock-ups.	KHumic	China
LIQHUMUS^®^ Liquid 18	14	4	liquid	Immediately improves plant nutrient absorption and fertilizer efficiency of soils; increases the stress tolerance of plants against drought, salt, cold and heat; stimulates vigorous root growth and yield formation; increases the buffering and cation exchange capacity of soils; acts as a natural chelator for micronutrients in soils and increases their availability to plants; stimulates the formation of fertile, microbially active soils; improves the soil structure and increases its water holding capacity; increases the germination of seeds and enhances development of free radicle.	Humin Tech	Germany
Prism Aqua Humic—Pure Crystal	60–65	15–20	powder	Extends the life of nano aquariums in particular; reduces stressful situations caused by high stocking density, transport, or high water temperatures; facilitates acclimatization; neutralizes organic poisons, unpleasant odours and the influence of foreign hormones; natural tannins and humic acids promote well-being and resistance; contains effective ingredients for preventing infectious diseases.	Prism	Germany
ROKOHUMIN Klasik	13	-	liquid	Increases the quality and intensity of plant respiration by better opening the stomata in the leaves; supports the immune system of plants; increases resistance to stress (weather, drought, temperature, excess light...); helps improve photosynthesis; has a beneficial effect on the development of the root system; preserves and saturates the natural taste of fruits and vegetables; restores and preserves the natural fertility of the soil; ecologically friendly and safe for animals and bees.	Humac^®^	Slovakia
Sodium Humate	70	-	powder	Removes toxic metals and their ions from wastewaters; an adjunct in dissolved air flotation cells to assist in the removal of trace amounts of grease, oil, liquid organics and suspended matter; a coagulant aid to be used in conjunction with water-soluble polymeric flocculants for removal of soluble organics; fluid loss additives in certain types of organic liquids to prevent seepage from lagoons or pit containment areas.	Mark Nature	China
Turbo Root	5.8	1.5	liquid	Roots: Humic and Amino Acid mix increases the number, and weight, of fine white roots and improves nutrient and water uptake by crops; increases the absorption of nutrients especially Phosphorus, Potassium and Zinc; increases the resistance of crops to stress from rapid growth and adverse climate; soil/potting substrate improves the structure and aggregation of soil encouraging root growth; increases the cation exchange capacity of soil which increases nutrient holding capacity; increase the population of beneficial microbes in the soil, promoting healthy soil.	Tradecorp	Spain

HA = Humic acids, FA = Fulvic acids.

## Data Availability

All data related to commercial products based on humic substances presented in this work come from sources that are publicly available.
